# Uncertainties around the Implementation of a Clearing-Control Policy in a Unique Catchment in Northern Australia: Exploring Equity Issues and Balancing Competing Objectives

**DOI:** 10.1371/journal.pone.0096479

**Published:** 2014-05-05

**Authors:** Vanessa M. Adams, Robert L. Pressey

**Affiliations:** 1 Northern Australia National Environmental Research Program Hub and Research Institute for the Environment and Livelihoods, Charles Darwin University, Darwin, NT, Australia; 2 Australian Research Council Centre of Excellence for Coral Reef Studies, James Cook University, Townsville, QLD, Australia; University of New South Wales, Australia

## Abstract

Land use change is the most significant driver linked to global species extinctions. In Northern Australia, the landscape is still relatively intact with very low levels of clearing. However, a re-energized political discourse around creating a northern food bowl means that currently intact ecosystems in northern Australia could be under imminent threat from increased land clearing and water extraction. These impacts are likely to be concentrated in a few regions with suitable soils and water supplies. The Daly River Catchment in the Northern Territory is an important catchment for both conservation and development. Land use in the Daly catchment has been subject to clearing guidelines that are largely untested in terms of their eventual implications for the spatial configuration of conservation and development. Given the guidelines are not legislated they might also be removed or revised by subsequent Territory Governments, including the recently-elected one. We examine the uncertainties around the spatial implications of full implementation of the Daly clearing guidelines and their potential effects on equity of opportunity across land tenures and land uses. We also examine how removal of the guidelines could affect conservation in the catchment. We conclude that the guidelines are important in supporting development in the catchment while still achieving conservation goals, and we recommend ways of implementing the guidelines to make best use of available land resources for intensified production.

## Introduction

The Earth is experiencing a new era, the Anthropocene, in which human actions have become the main driver of global environmental change, with many planetary boundaries being approached or already transgressed, and an already significant and accelerating loss of biodiversity [1]. Species extinction rates are estimated to be 100 to 1000 times their pre-human levels [2]. Human activities are the main drivers of greatly increased species extinctions, with land-use change being the most significant [3]. Despite a global commitment to protect biodiversity through the Convention on Biological Diversity, current indices show that biodiversity continues to decline while human pressures increase [4].

Typical approaches to addressing impacts of clearing of native vegetation on biodiversity are to set protection objectives so that minimum areas with priority for conservation are not cleared or, alternatively, to set limits such that clearing cannot exceed a specified level. In order for either approach to be effective in mitigating proximate threats to biodiversity, conservation planners must first be able to predict changes in the extent and intensity of threatening processes, such as land conversion, so that potential loss can be minimized [5], [6]; both conservation objectives and limits on clearing should be informed by potential clearing of particular ecosystems. Future patterns of land use, in terms of extent and rates of change in response to current and emerging driving forces, are not uniform within regions but can be understood with spatially explicit models [7]–[10]. If planners have spatially explicit data on potential future patterns of land-use change such as areas of high likelihood of clearing of native vegetation, these data can be used to minimize the loss of biodiversity by: 1) adjusting conservation objectives, 2) avoiding more threatened areas where there are spatial options, 3) selecting threat-specific actions, and 4) scheduling conservation actions [5], [11]. For example, scheduling acquisition of protected areas can minimize the extent to which conservation objectives are compromised by land clearing while the protected area system is being established [12]–[14].

Land-use change models identify areas of high likelihood of clearing and can be used to inform policies for protected areas or clearing controls that aim to reduce threats to biodiversity from clearing. However, standard approaches to land-use change modelling require historical data on clearing (typically at least 3 points in time to develop and validate the model) and assume that future clearing patterns will reflect the same driving factors as historical patterns [8]. These assumptions are unlikely to hold true for areas that have experienced low historical rates of clearing or that are experiencing changes in land-use drivers. Alternative methods of evaluating likelihood of clearing and implications for biodiversity conservation will therefore be needed in these cases.

Northern Australia contains nearly a third of the total global area of remaining tropical savanna [15] and is thus significant globally for savanna conservation. Much of Northern Australia remains sparsely populated with a relatively intact environment [15]. However, Northern Australia is often a focus of political discourse in Australia as a potential area for future development and expansion of agriculture [16], so future patterns and rates of clearing are likely to differ from historical trends. In particular, the Daly Catchment in the Northern Territory is an area of interest for future development due to a unique combination of suitable soils, year-round water supplied by large aquifers and the perennial Daly River, and suitable climatic conditions (adequate rainfall during the growing period) for rain-fed crops [17]. Current land use in the Daly River catchment is predominantly pastoral; however, there has been recent interest in clearing for both improved pastures and cropping. In response to concerns over potential clearing impacts on conservation values in the Daly catchment, the Northern Territory Government designed clearing guidelines that set limits on percentages cleared by property, vegetation type, sub-catchment, and the whole catchment (Table 1). The approach taken is unique in that it extends classic clearing-control approaches to include a number of nested hierarchical “caps”, thus termed the “cascade rules”, to ensure that clearing is distributed evenly across different biophysical features such as sub-catchments and vegetation types [18]. The approach ensures that overall clearing levels are controlled and that clearing does not substantially reduce vegetation types in areas attractive to clearing.

**Table 1:** Summary of cascade rule caps for clearing specified in the clearing guidelines for the Daly catchment [18].

Feature	Percentage clearing cap
Streams – 250 m buffer	Clearing prohibited^a^
Wetlands – 250 m buffer	Clearing prohibited^a^
Daly River – 1000 m buffer	Clearing prohibited^a^
Rainforest – 250 m buffer	Clearing prohibited^a^
Property	70%
Sub-catchment	40%
Vegetation type	30%
Catchment	20%

a Buffer zones have generally been supported by the process of assessing clearing applications. However, requests from landholders for exceptions could be approved, with the risk of buffer zones being reduced in unpredictable ways. Neither the extent nor the distribution of ad hoc clearing in buffer zones could be modelled for this study.

This is an interesting clearing-control approach that could be considered more broadly for protection of native vegetation across northern Australia and internationally. However, the eventual outcome of the restrictions, when all clearing opportunities have been taken, is likely to be sensitive to the order in which properties take up clearing options. The approach therefore needs to be tested more thoroughly to ensure against perverse outcomes. For example, if a sub-catchment reaches the allowed clearing because one or two large properties have used their clearing options, then the remaining properties in the sub-catchment cannot clear.

Understanding the different ways in which clearing guidelines in the Daly catchment could unfold, given scope for extensive further clearing under the cascade rules, has important implications for land management generally and conservation management specifically. The sequence with which properties are cleared is affecting and will further affect equity of opportunity between grazing and horticultural enterprises. This is particularly relevant considering that 26% of the land available for clearing in the catchment is Indigenous land (land either held or managed by Indigenous Australians) without immediate plans for development. Additionally, predicted patterns of clearing flowing from the cascade rules can inform recommendations on the most important areas to prioritize for development to ensure that areas of high production value are developed within clearing limits. Conversely, if areas of high conservation value are identified, the same predictions can be used to prioritize those that are also vulnerable to clearing.

Uncertainties around eventual clearing patterns in the Daly catchment were widened by a change of government in the Northern Territory in September 2012. The new Government is developing new policies for water resources [19] and, given the clearing guidelines are not legislated, the new Government, or subsequent Governments, could revise the cascade rules or replace them. It is therefore critical to understand the potential of existing policies such as the clearing guidelines to contribute to conservation objectives and to explore potential clearing patterns for the catchment in the absence of any guidelines.

Currently, only about 5% of the Daly Catchment has been cleared but a lack of pre-clearing vegetation mapping means we cannot interpret clearing by vegetation structure or assess previous losses by vegetation type. Furthermore, previous clearing is unlikely to reflect future clearing patterns given the change in political focus toward agricultural development and significant changes in policies for land and water resources. Additionally, the cascade clearing guidelines have the potential to constrain future clearing, even in areas suitable for development, so standard methods for land-change modelling are not appropriate. Our study takes an alternative approach to modeling future clearing patterns in the Daly catchment with three main objectives:

- To develop maps of potential clearing for use in regional planning to prioritize areas for competing objectives, namely development and conservation;
- To explore the potential influences of the clearing guidelines on spatial patterns of clearing and equity of opportunity to clear between different land uses and tenures;
- To explore the potential extent and pattern of clearing that could occur if the clearing guidelines were removed, as compared to potential clearing under the guidelines explored in 2.

We analysed scenarios to simulate patterns of clearing under varying assumptions, with and without the cascade rules. This approach allowed us to identify the sensitivity of potential clearing patterns to factors such as land suitability for pastoral and agricultural uses, land tenure, and property size and to make recommendations on implementation of the guidelines to ensure that clearing opportunities are equitable between stakeholder groups across the catchment.

## Materials and Methods

### Study region

**Figure 1:**
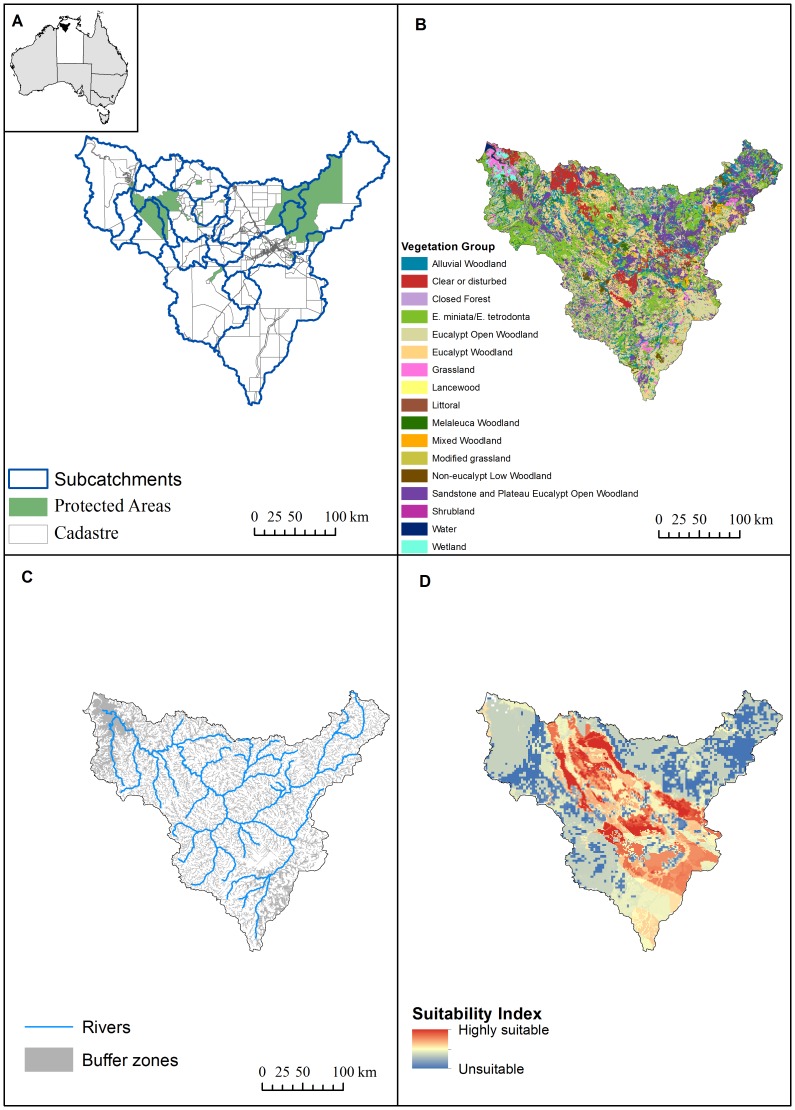
Daly River catchment. Inset in panel A shows Australian states in grey, the Northern Territory in white and the Daly catchment in black. A. Daly River catchment, property boundaries, protected areas (National Parks, such as Nitmiluk Gorge in the northeast, Conservation Areas and Indigenous Protected Areas, such as Fish River in the northwest) and sub-catchments. B. Fifteen vegetation groups based on a hierarchical classification. The cascade rules relate to 98 vegetation types mapped as subdivisions of these groups. C. Major rivers and buffer zones along streams, rivers, sinkholes and wetlands. D. Suitability index. The index indicates suitability of land for pastoral and agricultural clearing. Suitability ranges from zero (unsuitable, shown in dark blue) to highly suitable (400). Highly suitable land is appropriate for multiple land uses including modified pastures, rainfed and irrigated crops, and perennial and annual horticulture.

The study region was the whole of the Daly River catchment in the Northern Territory, which is approximately 5.2 million ha, extending from the coastline south-west of Darwin to 250 km inland (Figure 1A). The Daly River catchment has substantial conservation values, including five sites of conservation significance identified by the Northern Territory Government [20], extensive gallery rainforest, and habitats for important wildlife populations, especially of fish, turtles, and waterbirds. The sites of conservation significance within the Daly River catchment have been assessed as either nationally or internationally significant. Although there are no Ramsar-listed wetlands within the catchment, Chatto [21] noted that the Daly River estuary and lower floodplain are likely to qualify for Ramsar listing based on waterbird numbers. More recently, additional studies have identified areas such as the Daly middle reaches and floodplain as high conservation priority [22], [23]. Approximately 13% of the catchment is protected by national parks, such as Nitmiluk Gorge, and Indigenous protected areas (Indigenous-owned land enrolled in the Australian national reserve system with the purpose of promoting conservation of biodiversity and cultural resources) such as Fish River (Figure 1A). Of the area potentially available for clearing (87% of the catchment), 10% is government held, 30% is aboriginal land, and 60% is private property (predominantly pastoral). The average size of private properties in the Daly is ∼10,500 ha, with properties larger than 5,000 ha representing approximately 13% of landholders but about 90% of the catchment's private land.

### The cascade rules

The Daly catchment has been recognized both for its high conservation values as well as its potential for further development. Therefore, although only 5.4% of the catchment has so far been cleared, in 2010 the Northern Territory Government designed clearing guidelines in the form of the cascade rules in response to expected pressure for clearing. Major aims of the approach are to ensure that areas suitable for development are available for clearing while areas of conservation value are adequately protected [18]. The cascade rules involve clearing “caps” specified for features defined at different resolutions (Table 1). When a cap is reached at any level, future clearing is precluded for that feature. The clearing guidelines specify that no more than 20% of the catchment area can be cleared. The cascade rules are supported by vegetation mapping across the catchment (Figure 1B) and there are designated buffer zones around sensitive habitats, such as streams and rivers, in which no clearing is allowed (Figure 1C).

Under the clearing guidelines, all landholders of properties greater than 100 ha must submit a clearing application adhering to the cascade rules. Landholders of properties under 300 ha in size can submit clearing applications without an environmental impact assessment (EIA), while landholders of properties larger than 300 ha must have an EIA before an application will be approved. EIAs can be costly and might therefore deter larger properties from submitting requests to clear. While aboriginal land comprises over one quarter of the catchment, there are no known development plans on this tenure. In comparison, many private landholders have expressed an interest in immediate clearing for intensive land uses.

Because of the nested nature of the caps, the order of clearing and the size and location of cleared properties can preclude other properties from clearing. For example, properties subject to the clearing guidelines range in size from 100–400,000 ha with an average size of 15,000 ha. This means the largest property could singly clear up to 5% of the catchment (and 25% of the available cap) by exercising the right to 70% clearing (assuming no other caps are met within the property). Furthermore, depending on the number, size and tenure of properties within a sub-catchments and vegetation types, properties could be excluded from clearing because neighbouring properties clear up to the sub-catchment or vegetation-type limit.

Aside from potential inequities between tenures and properties, the implications of clearing caps for sub-catchments and vegetation types are difficult to anticipate. Even though all sub-catchment caps are the same (Table 1), sub-catchments vary widely in tenure, including percentages in conservation reserves (Table 2), so the potential for and implications of clearing vary widely between sub-catchments. Similarly, caps for all vegetation types are the same, although vegetation types vary in conservation status and threats beyond the Daly catchment, in conservation status of associated species, and possibly in spatial turnover of species. So the implications of clearing are also likely to vary between vegetation types. Unfortunately, species mapping for the region is limited so it is difficult to properly assess the relative importance of different vegetation types for conservation of individual species. Future improvement in species data would contribute to a more in-depth understanding of whether variable clearing caps would be appropriate and whether clearing should avoid localised areas of particular value to certain species.

**Table 2:** Sub-catchment details.

Sub-catchment	Number of properties	Reserve	Aboriginal
Daly River	86	9.05%	50.04%
Chilling Creek	6	1.71%	11.95%
Hayward Creek	4	0.53%	0.00%
Fish River	5	40.08%	71.70%
Bamboo (Moon Boon) Creek	3	53.04%	100.00%
Green Ant Creek	8	0.00%	0.00%
Douglas River	26	2.12%	1.56%
Stray Creek	20	3.46%	4.43%
Bradshaw Creek	7	0.00%	50.31%
Dead Horse Creek	5	0.00%	27.86%
Fergusson River	66	20.38%	54.19%
Flora River	17	1.11%	37.33%
Katherine River	156	32.09%	74.61%
Limestone Creek	4	0.00%	0.00%
King and Dry Rivers	52	2.03%	4.80%
Seventeen Mile Creek	8	100.00%	100.00%

Number of properties, percentage reserved by area and percentage of aboriginal land by area are given for each subcatchment.

Another potential risk from applying the cascade rules is that, depending on the sequence of clearing approvals below the caps in Table 1, clearing of land with low to moderate production value could pre-empt development of areas with high production value.

### Data

To conduct our analysis we used the cadastre for the catchment to define property boundaries (Figure 1A). We used vegetation mapping for the Daly river catchment in order to calculate existing percentage cleared and current extent of vegetation types [24]. This vegetation mapping has 98 vegetation types mapped and attributed to 15 broad scale vegetation groups Figure 1B). However, this vegetation mapping does not have an associated pre-clearing product. Therefore, in order to estimate current clearing by vegetation types we used the vegetation groups specified within the mapping and related to the pre-clearing product available for these broad groups [25] to calculate current percentage cleared. We assumed that clearing levels were uniform across vegetation types associated within a broad vegetation group. We mapped the buffer zones using spatial maps of streams, rivers, wetlands and sinkholes (Figure 1C).

As a guide to possible future patterns of clearing, and to help understand the effects of different clearing sequences on development potential in the catchment, we created a land suitability index. We based our index on land suitability mapping completed by Pascoe-Bell et al. [17] which assessed land suitability using available data on land systems, soil, slope, rainfall, and access to ground water and surface water. Land suitability was mapped as a percentage of land system (0–100%) suitable for four land uses: improved pasture (includes introduced pasture species, usually grasses in combination with legumes), irrigated field crops and perennial horticulture, irrigated annual horticulture, and rainfed field crops and perennial horticulture [17]. For our study, we created an overall clearing suitability index that summed the suitable percentage of each land system across the four land uses (Figure 1D). Our index ranged from 0 – 400, with 0 being completely unsuitable across all land uses (dark blue in Figure 1D) and 400 being completely suitable across all land uses. Although summing values for individual uses gave values larger than 100% for some land systems, we assumed that the range of values larger than 100 indicated relative demand for clearing due to production potential and flexibility of uses.

### Simulation of catchment-wide clearing sequences subject to clearing guidelines

The cascade rules were released in 2010 and have only recently been implemented in clearing permits. Very few caps have been reached because only 5.4% of the catchment has been cleared; therefore the guidelines' influence on spatial patterns of clearing is not well understood. We designed four scenarios that allowed us to investigate the influence of different sequences of clearing on the spatial patterns of clearing as well as well as relative amounts of clearing across land tenures. When designing the scenarios we took into account factors such as tenure (aboriginal land versus private properties), size of property, and average suitability of land. The four scenarios were:

- Random – Properties were selected in a random order. We use this as our baseline for comparison with the other scenarios guided by the cascade rules.
- Non-aboriginal, large properties first – Properties were selected from largest to smallest with non-Aboriginal properties clearing first. This scenario reflects the apparent lack of plans to clear aboriginal properties over the short term and the assumption that larger properties would have more financial resources to complete the required EIAs.
- Non-aboriginal, small properties first – Properties were selected from smallest to largest with non-aboriginal properties clearing first. This scenario reflects the apparent lack of plans to clear aboriginal properties over the short term and the assumption that EIAs could be a barrier to larger properties clearing.
- Directed clearing – Properties were selected based on clearing suitability averaged across the property, in descending order. This scenario reflects the case in which clearing is guided to maximize the use of the most suitable land within the caps.

**Figure 2:**
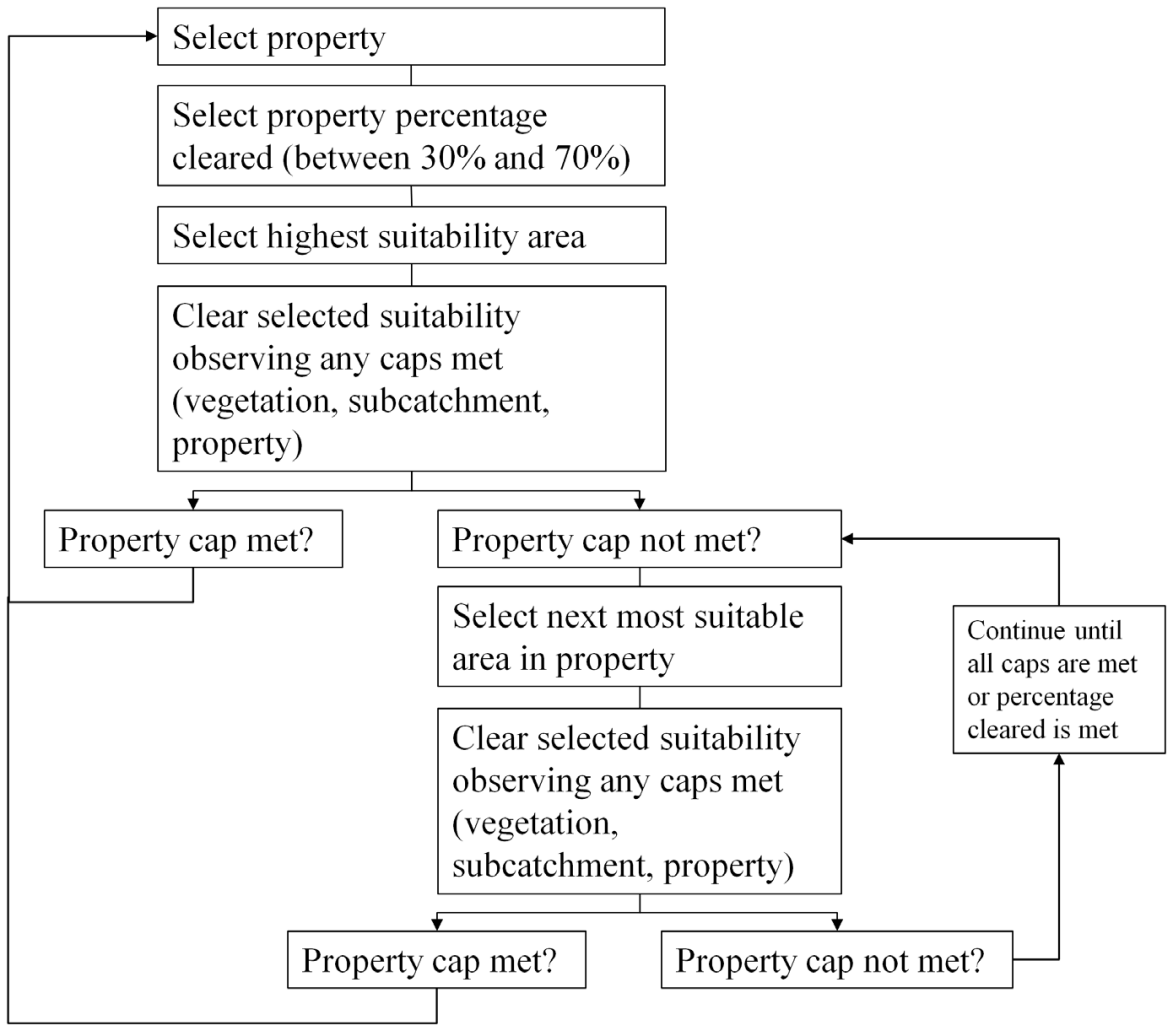
Algorithm for simulating clearing of properties based on cascade rules.

We simulated clearing for each scenario by selecting the property order based on the rules described above and implementing an algorithm for clearing within the cascade rules (Figure 2). Properties with existing clearing have percentage areas cleared ranging from 1–100% with an average of 25%. Given this historical clearing is unlikely to reflect changing land uses and therefore changing patterns of clearing, we used a lower bound of property clearing of 30% and the upper bound of 70% specified by the guidelines. For each selected property we identified a percentage level of clearing based on a uniform distribution between 30% and 70%. We assumed that property owners would first clear land most suitable for production to maximize profits. Within each selected property, areas with the same suitability index were intersected with vegetation types such that the property was divided into units of unique combinations of suitability and vegetation type. For each property, we sequentially simulated clearing of areas in order of decreasing suitability, clearing 100% of each unit with the highest suitability if no caps had been reached and otherwise taking into account caps imposed by previous clearing of vegetation types and sub-catchments until we reached the identified percentage of the property to be cleared, or until all available suitable land (suitability >0) had been cleared if this was less than the identified percentage. We followed this stepwise process for each property until the catchment-wide clearing limit of 20% was reached. Because of the stochastic allocation of clearing percentages to properties, even in scenarios 2–4, we ran each scenario 100 times.

### Simulation of catchment-wide clearing sequences with no clearing guidelines

When considering only land suitable for clearing (all areas except those classified as ‘unsuitable’ or suitability  = 0 in Figure 1D) outside of buffer zones and currently protected areas, about 2.3 million ha or 44% of the catchment could be cleared. This means that, in the absence of the cascade rules and assuming that future clearing will occur only on suitable land, a total of 49.4% of the catchment might eventually be cleared (44% potential and 5.4% existing).

Given the guidelines are not legislated and could therefore be relaxed or removed, we investigated the extent and potential spatial patterns of unconstrained clearing by running a clearing simulation without any caps related to vegetation types, sub-catchments, or the whole catchment. We used the random ordering applied in Scenario 1 but retained the uniform distribution of property clearing from 30–70% to reflect the likelihood that most properties will retain some vegetation around residences and for grazing of livestock on native pastures (pastures dominated by native plant species) - a major land use currently in the catchment. We also retained the buffer areas because they reflect best-practice clearing recommendations outside of the guidelines for preventing erosion and maintaining water quality. We ran the simulation 100 times. The worst-case scenario of clearing with no guidelines, assuming that landholders are profit-driven and would not invest in clearing unsuitable land with little promise of financial returns, is given by our calculation above of 49.4% of the catchment. Our simulation in the absence of guidelines constrains this potential clearing somewhat by assuming variable clearing percentages across properties.

### Simulation Results

For each scenario - four constrained by the cascade rules and one unconstrained - we calculated the average percentage cleared for each vegetation type on each property across the 100 runs. We also calculated the average percentage area cleared by sub-catchment and by vegetation type across the entire catchment. For properties that cleared land, we recorded their number, tenure, average suitability, and average size.

## Results

**Figure 3:**
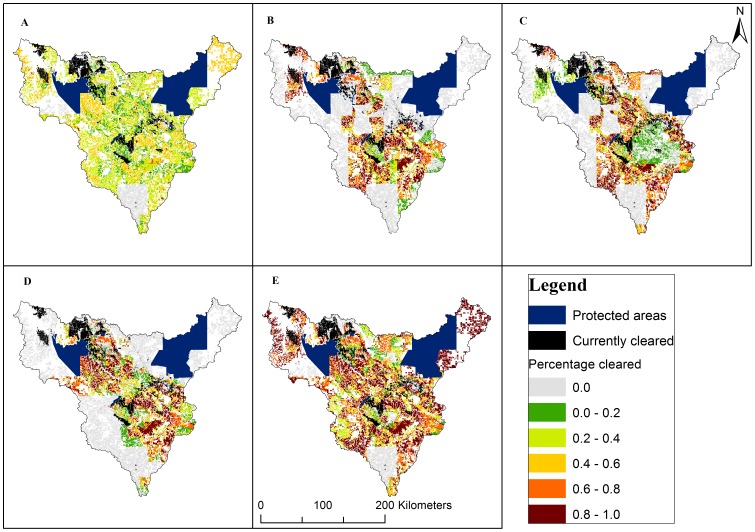
Patterns of clearing from simulations for five scenarios. Percentages cleared are shown by vegetation type within property stratified by land suitability. Currently cleared areas are shown in black and protected areas are shown in blue. Areas available for clearing but not cleared are in pale gray and areas not available for clearing due to being within a buffer zone or unsuitable (index = 0) are in white. A. Scenario 1 – Random, constrained by cascade rules. B. Scenario 2 - Non-aboriginal, large properties first, constrained by cascade rules. C. Scenario 3- Non-aboriginal, small properties first, constrained by cascade rules. D. Scenario 4 – Directed clearing, constrained by cascade rules. E. Scenario 5 – Random, not constrained by clearing guidelines.

The four scenarios constrained by the clearing guidelines and the single scenario ignoring the clearing guidelines produced markedly different spatial patterns of clearing across the catchment (Figure 3). For example, scenario 1 (random) resulted in moderate levels of clearing across the entire catchment (Figure 3A) while scenario 4 (directed clearing) resulted in high levels of clearing constrained to the areas of highest suitability in the catchment (Figure 3D, and compare Figure 1D). The potential influence of the clearing guidelines on the overall magnitude of clearing can be seen by comparing scenario 1 (random, constrained) with scenario 5 (random, unconstrained). While both scenarios result in widespread clearing, the overall amount of clearing in scenario 5 (>1.7 million ha of new clearing resulting in a total of 38% of the catchment cleared) is more than double that in scenario 1 and in the other constrained scenarios (Table 3) because all properties that are open for clearing take that opportunity.

**Table 3:** Summary statistics related to properties, by scenario.

	Scenario 1 – Random	Scenario 2 – Large first	Scenario 3 – Small first	Scenario 4 - Directed	Scenario 5 - No guidelines
Average number of properties with clearing	120	19	228	131	270
Average size (ha) of properties with clearing	18,104	109,930	9,273	15,961	17,140
Average total area cleared (ha)	799,134	774,676	787,104	772,242	1,710,709
Average area cleared - aboriginal properties (ha)	339,531	0	0	164,852	661,155
Average area cleared - non-aboriginal properties (ha)	459,603	774,676	787,104	607,390	1,049,554
Average property suitability (index 0–400)^a^	171	167	180	227	172

a Average property suitability is the average suitability across all land on properties selected for clearing. The minimum average property suitability was 3.6 and the maximum was 301. The average suitability across all properties in the Daly catchment was 178.

The average size of property that cleared and the total area cleared varied across scenarios. The assumption that large properties cleared first (Scenario 2) resulted in substantially fewer, much larger properties with clearing (Table 3). This strong difference reflects large variation in property sizes across the catchment. The largest properties in the catchment exceed 100,000 ha. If these large properties all clear large percentages of their holdings, the total available area for clearing across the catchment can be reached with clearing on 15– 27 properties. The sequence with small properties clearing first (scenario 3) led to many more properties having opportunities to clear.

The effect of land tenure in the clearing sequence is evident in scenarios 2 and 3. In both scenarios, opportunities for clearing across the catchment were exhausted on non-aboriginal land before any aboriginal properties had the opportunity to clear (Table 3). In the guided scenario 21% of land cleared was aboriginal, slightly below the overall percentage of aboriginal land (26% of available land). In scenarios that ignored tenure and land quality (1,5), a larger proportion of aboriginal properties were cleared (scenario 1 – 42.5% of total clearing; scenario 5 – 38.6%).

As expected, the directed clearing of scenario 4 selected properties with the highest average suitability (227, Table 3). The random scenarios (1 and 5) produced average property suitabilities somewhat below the catchment-wide average of 178. The lowest average property suitability was 167 for scenario 2 (large properties first), reflecting the larger proportion of land on these properties classified as suitable only for modified pastures and not for other agricultural uses.

Differences in spatial clearing patterns were also evident in the average percentages of sub-catchments cleared (Table 4). There was a large effect of land tenure. For example, Bamboo Creek sub-catchment, totally within aboriginal tenure (Table 2), remains totally uncleared in scenarios 2 and 3 that allocate no clearing to aboriginal land, but is moderately cleared in other scenarios. Differences between scenarios 2 and 3 in terms of percentages of sub-catchments cleared relate to the distribution across sub-catchments of properties of different sizes. Directed clearing (scenario 4) resulted in lower levels of clearing in sub-catchments dominated by low-suitability land, such as Chilling Creek, and higher levels of clearing in highly suitable sub-catchments regardless of tenure, such as Bradshaw Creek and Limestone Creek. As expected, most sub-catchments were more extensively cleared in scenario 5, without clearing guidelines, compared to scenario 1.

**Table 4:** Average percentage clearing by sub-catchment, current and by scenario.

Sub-catchment	Current	Scenario 1 – Random	Scenario 2 – Large first	Scenario 3 – Small first	Scenario 4 - Directed	Scenario 5 - No guidelines
Daly River	7.91%	21.03%	18.35%	16.03%	21.17%	37.46%
Chilling Creek	1.51%	11.37%	16.28%	4.87%	2.41%	25.55%
Hayward Creek	4.02%	11.84%	19.61%	8.86%	15.88%	21.25%
Fish River	0.00%	5.37%	0.00%	0.00%	10.47%	10.76%
Bamboo (Moon Boon) Creek	0.00%	13.02%	0.00%	0.00%	24.23%	25.75%
Green Ant Creek	60.35%	60.35%	60.35%	60.35%	60.35%	60.35%
Douglas River	18.39%	30.42%	35.95%	39.24%	36.52%	41.30%
Stray Creek	10.15%	25.87%	36.85%	37.64%	36.89%	40.66%
Bradshaw Creek	0.94%	20.43%	24.11%	24.85%	35.64%	39.91%
Dead Horse Creek	2.92%	23.94%	35.56%	36.37%	38.28%	41.83%
Fergusson River	1.44%	20.98%	11.80%	22.75%	12.55%	40.50%
Flora River	3.57%	22.89%	29.73%	29.29%	11.50%	42.56%
Katherine River	4.48%	19.34%	8.12%	10.06%	9.87%	37.93%
Limestone Creek	16.32%	28.23%	37.70%	26.16%	37.16%	39.54%
King and Dry Rivers	0.94%	18.03%	25.35%	24.48%	29.54%	38.73%
Seventeen Mile Creek	0.00%	17.78%	0.00%	0.00%	0.00%	42.50%
Total	5.41%	20.68%	20.22%	20.45%	20.17%	38.10%

**Figure 4:**
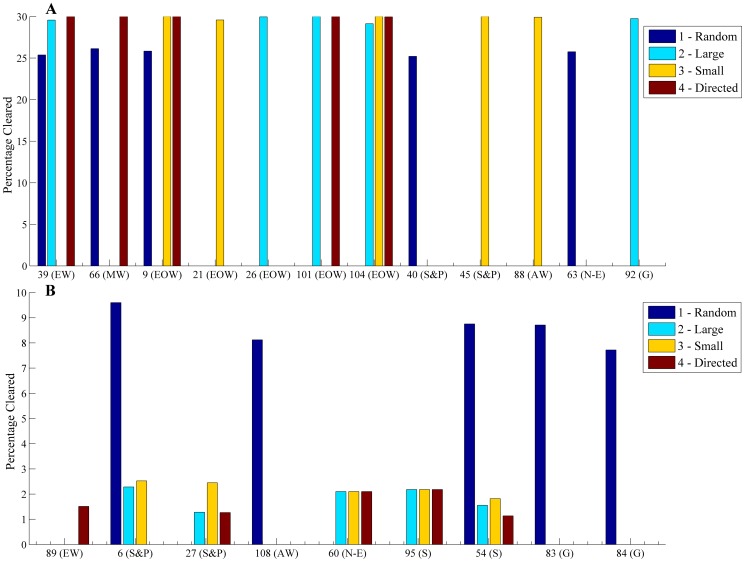
Vegetation types with the highest and lowest levels of clearing in the clearing guideline scenarios. A) Vegetation types with highest clearing in guideline scenarios (1–4). The top five cleared vegetation types in one or more scenarios are shown with percentages cleared by scenario. B) Vegetation types with lowest clearing in guideline scenarios (1–4). The bottom five cleared vegetation types in one or more scenarios are shown with percentages cleared by scenario. Note differences in scale of *y*-axes. Vegetation codes and names of broad vegetation group are displayed^ a^ [24]. ^a^ Vegetation groups are shown in Figure 2B. Closed Forest (CF), Eucalypt Woodland (EW), Mixed Woodland (MW), E. miniata/E. tetrodonta (E. min/E. tet), Eucalypt Open Woodland (EOW), Sandstone and Plateau Eucalypt Open Woodland (S&P), Lancewood (L), Melaleuca Woodland (MelW), Alluvial Woodland (AW), Non-eucalypt Low Woodland (N-E), Shrubland (S), Grassland (G), Littoral (Li).

The average percentage cleared by vegetation type was similar across scenarios 1–4, and clearing was on average about twice as much by vegetation type without clearing guidelines (scenario 5) (Table 5). For each scenario we compared the percentage area cleared for each vegetation type. Across all vegetation types, there were strong positive correlations between scenarios in terms of percentage clearing across all 98 vegetation types, with *ρ* ranging from 0.6491 to 0.9165 (Table 6). Scenarios 1 and 5, with random selection of properties, were most similar (*ρ* = 0.9165). This was expected because the only difference between these two scenarios was the limit on total clearing. The next most similar scenarios were 2 and 3 (*ρ* =  0.8945), both constrained to non-aboriginal land, indicating that clearing small properties first (scenario 3) results in only a small percentage of catchment-wide clearing, allowing substantial scope for subsequent clearing on larger properties, similar to that in scenario 2. However, there were differences in the levels of clearing within individual vegetation types. Scenarios 1–4 had highly variable impacts on the vegetation types most affected by clearing (Figure 4A), but Scenarios 2–4 targeted similar levels of clearing for the least cleared vegetation types (Figure 4B). Scenario 1 had overall higher levels of clearing across the least-cleared vegetation types due to its more dispersed pattern of clearing.

**Table 5:** Average percentage cleared and standard deviation across vegetation types (n = 98), by scenario.

	Scenario 1 - Random	Scenario 2 – Large first	Scenario 3 – Small first	Scenario 4 - Directed	Scenario 5 - No guidelines
Average percentage cleared by vegetation type	16.81%	14.51%	15.32%	12.68%	32.48%
Standard deviation of percentage cleared by vegetation type	6.02%	8.82%	8.65%	8.67%	14.50%

**Table 6:** Spearman's Rank Correlation Coefficient (ρ) between pairs of scenarios in terms of percentage vegetation type cleared (n = 98).

	Scenario 1	Scenario 2	Scenario 3	Scenario 4	Scenario 5
Scenario 1	1				
Scenario 2	0.7020	1			
Scenario 3	0.7245	0.8945	1		
Scenario 4	0.7168	0.8355	0.8139	1	
Scenario 5	0.9165	0.6491	0.6451	0.6615	1

All p<0.001.

## Discussion

The clearing guidelines constrained total clearing in the catchment to 20% compared to a potential maximum of 49.4% if all available suitable land were cleared and a simulated 38% in our unconstrained scenario, considering realistic percentages of properties cleared. A policy shift to removing the cascade rules, along with other perceived impediments to development in the Daly catchment, could expose some vegetation types and their associated flora and fauna to high levels of clearing. Extensive clearing of the Daly catchment or other parts of northern Australia involves substantial risks for biodiversity. Broad-scale grazing and changed fire regimes associated with intensified land uses have been implicated in declines in small mammals and granivorous birds in northern Australia [26]–[28]. Furthermore, invasive species originally introduced for improved pastures pose a significant threat to both environmental and economic values [29]. These risks to the Daly's biodiversity apply even with the cascade rules in place. While the guidelines ensure that 80% of the catchment will remain uncleared, and riparian habitat is protected in buffer zones, they still allow significant clearing of some properties and sub-catchments, with poorly understood implications for conservation. In addition, the impacts of land clearing extend beyond the loss of vegetation. For example, land clearing and associated development is often associated with the introduction of invasive species and changed fire regimes whose impacts extend well beyond the cleared sites. Policy makers should carefully monitor and regulate these impacts as clearing proceeds.

The cascade rules have been criticized for lack of flexibility to accommodate development in the catchment. However, our directed clearing scenario demonstrates that the guidelines can allow for clearing of highly suitable land for intensification or expansion of agriculture or pastoralism (scenario 4). Thus, the cascade rules, if implemented carefully, present one policy approach to addressing competing land use objectives of conservation and development. Our analyses also indicate that, even in the absence of the clearing guidelines but with realistic percentage clearing of properties, cleared areas by sub-catchment are typically about 42% or less. So the 40% sub-catchment cap in the cascade rules will not substantially constrain clearing of suitable land or preclude clearing by property managers who wish to develop.

The catchment contains the Daly Basin bioregion in its entirety as well as portions of several other bioregions. While we used the best available vegetation mapping for our analysis, this mapping does not reflect compositional and fine-resolution structural variation in vegetation types within and between bioregions in and around the catchment. Implementing the cascade rules with the existing vegetation mapping could therefore result in loss of biodiversity, including critical habitat for fauna. For example, a directed application of the guidelines (scenario 4) would focus clearing on highly suitable land occurring mainly within the Daly Basin bioregion and minimize clearing in other bioregions intersecting the catchment [30]. A better understanding of species distributions in the catchment is needed to accurately identify areas of high conservation value. If this data were available, clearing scenarios could be used to identify areas of both high conservation value and high vulnerability to clearing, leading the way to informed tradeoffs between conservation and development goals.

Given the nested nature of the clearing guideline caps, the order in which clearing occurs can strongly influence the spatial pattern of clearing across the catchment. We found that a random order of property clearing with the guidelines produced similar spatial patterns to clearing without guidelines, demonstrating that the guidelines do not inherently bias clearing to any portion of the catchment. However, the guidelines have the potential to preclude clearing by aboriginal properties if managers of these properties delay clearing (scenarios 2 and 3). Importantly, the directed scenario focused on high-quality land cleared a representative proportion of aboriginal and non-aboriginal land, allowing for equitable access to property development across land tenures. Scenario 2 showed that, if the largest properties clear first, much of the highly suitable land in the current agricultural and horticultural zones, such as the Douglas Daly, will remain uncleared under the guidelines. The predominant features of cleared land on large properties are modified and improved pastures for grazing (including a current application for 18,000 hectares for grazing on one of the largest properties in the Daly) [31]. If clearing occurs on large properties first, then the 20% of allowed clearing across the catchment would be allocated mainly to cattle grazing at the expense of agriculture and horticulture.

Overall, it is clear that the sequence of clearing under the cascade rules could influence equity of opportunity to develop between aboriginal and non-aboriginal properties, and could determine the relative extents of grazing and horticulture. This result highlights the need for a policy surrounding the implementation of the guidelines to ensure equity and balance between tenures and uses. One implementation policy that could accompany the guidelines is a directed or zoned approach in which areas that are identified as highly suitable for development are earmarked as available for clearing. For example, our directed clearing scenario led to almost proportional clearing across tenure types, so this strategy would address equity issues while insuring that the most suitable land was available for clearing. Another implementation policy could be to mirror the approach taken in water allocations in which a Strategic Indigenous Reserve (SIR) is included in the allocation to ensure a proportional allocation of water is held for aboriginal development. A SIR could be included in the caps such that an equitable portion of the 20% clearing cap is reserved for clearing on aboriginal properties.

The political discourse around a northern Australia ‘food bowl’ to supply Asia has recently reignited, with consideration of removing the Daly's cascade rules and a recent announcement by the Queensland Government to release new water licenses in the North for crop production [32]. Similarly, Western Australia has announced plans for Stage 2 of the Ord River irrigation scheme and an announcement is expected from the Northern Territory Government about further expansion into Ord Stage 3 [33]. This push for development across relatively intact savanna landscapes [15] comes with risks to biodiversity that are not well understood. The available data, however, indicate continuing declines of many species [28] and offer nothing to support optimism about the coexistence of northern Australian biodiversity with expanding transformation of landscapes [27]. In this context, and even with uncertainties about their conservation implications, the Daly's cascade rules are precautionary while still providing opportunities for development. With adjustments for equity of opportunity and balanced land uses, and with refinements to ensure that the best ecological data are considered in setting limits to clearing in specific parts of the catchment, the Daly clearing guidelines provide a model for other regions across the northern savannas and beyond.
